# New Composite Water Sorbents CaCl_2_-PHTS for Low-Temperature Sorption Heat Storage: Determination of Structural Properties

**DOI:** 10.3390/nano9010027

**Published:** 2018-12-26

**Authors:** Alenka Ristić, Nataša Zabukovec Logar

**Affiliations:** 1 Department of Inorganic Chemistry and Technology, National Institute of Chemistry Slovenia, Hajdrihova 19, SI-1001 Ljubljana, Slovenia; natasa.zabukovec@ki.si; 2 School of Science, University of Nova Gorica, Vipavska 13, 5000 Nova Gorica, Slovenia

**Keywords:** CaCl_2_-PHTS, composites, water sorption, heat storage, structural properties

## Abstract

Sorption heat storage, as one of low-energy consuming technologies, is an approach to reduce CO_2_ emissions. The efficiency of such technology is governed by the performance of the applied sorbents. Thus, sorbents with high water sorption capacity and regeneration temperature from 80 to 150 °C are required. Incorporation of hygroscopic salt such as calcium chloride into porous materials is a logical strategy for increasing the water sorption capacity. This work reports the study on the development of composites with PHTS (plugged hexagonal templated silicate) matrix with an average pore size of 5.7 nm and different amounts of calcium chloride (4, 10, 20 wt.%) for solar thermal energy storage. These composites were prepared by wetness incipient impregnation method. Structural properties were determined by X-ray diffraction (XRD), nitrogen physisorption, scanning electron microscopy (SEM) and transmission electron microscopy (TEM). CaCl_2_ was confined in micro- and mesopores of the matrix. The resulting CaCl_2_-PHTS materials were used for water sorption at 40 °C, showing an increase of maximal water uptake with higher amount of calcium chloride from 0.78 g/g to 2.44 g/g of the dry composite. A small reduction in water uptake was observed after 20 cycles of sorption/desorption between temperatures of 140 °C and 40 °C, indicating good cycling stability of these composites under the working conditions.

## 1. Introduction

Thermal energy storage (TES) is becoming a crucial technology in enabling more efficient use of renewable energy and contributing to the reduction of our dependency on fossil fuels. It can be divided into three main categories according to how energy is stored: sensible heat (e.g., water tanks, underground storage), latent heat (e.g., ice, phase change materials), and thermochemical heat storage [1]. Thermochemical heat storage utilizes the reversible chemical reaction [2] and/or sorption processes [3] involving working fluids and solids or liquids. Sorption thermal energy storage depends on thermo-physical properties of the sorbents such as thermally stable microporous and mesoporous materials. The main criteria for the selection of a proper sorbent for sorption thermal energy storage are high sorption capacity, low desorption temperature, and high temperature level of released heat of adsorption [4]. A large number of sorbents are currently considered for sorption thermal energy storage, traditional such as silica gels and zeolites, and innovative like aluminophosphates, MOFs, and composites [5,6]. The most versatile class of sorbents are the two-component sorbents or composite salt in porous inorganic matrix (CSPM) [7] which combine the advantages of the pure porous matrix and hygroscopic salt hydrates for the enhancement of water sorption capacity, heat, and mass transfer on one side, and on the other hand to avoid or reduce the deliquescence and agglomeration of salt hydrates during sorption/desorption cycles. The composites developed thus far can be used also with other working fluids, such as methanol [8]. The sorption properties of the composites can be tailored by varying chemical nature, amount, and particle size of the incorporated salt and depend strongly on the structural and physico-chemical properties of the porous host matrix (pore size/shape, pore volume, and hydrophilic properties) and host-salt interactions. Further advantages are a low desorption temperature, a low price and a simple preparation method [9]. Besides wet impregnation and incipient wetness impregnation procedures, microencapsulation is another approach for the stabilization of salt hydrates by enveloping with a second inherently stable material to prevent coalescence or agglomeration [10].

As a safe, environment-friendly, and available sorbate, water is usually the preferred choice. A combination of three mechanisms, adsorption by the host matrix, chemical reaction between water and salt, and absorption by the salt aqueous solution in the pores, determines water sorption behavior of the composites [11]. Typical hygroscopic salt hydrates incorporated in the composites for sorption TES are LiCl, MgCl_2_, CaCl_2_, MgSO_4_, and SrBr_2_ [1]. CaCl_2_ has been combined several times with different inorganic host materials such assilica gels [12], disordered mesoporous iron silicate [13], ordered mesoporous silicas [14], mesoporous alumina-silica [15], Ca-exchanged binder-free zeolite X [16], alumina [17], clays [18], MOFs [19], and carbonaceous structures [20] because of its low cost, non-toxicity, large availability, and a high sorption capacity [21]. In general, the role of the matrix is to adsorb water and serve as a dispersion medium, which forms a required salt particle size and high salt surface area. In addition, this prevents forming of typical agglomeration of salt particles and conducts heat through the solid. Usually, the porous matrix has lower water sorption capacity than the hygroscopic salts, which interact with water to increase the sorption capacity [11].

Mesoporous ordered silicates, MCM-41 and SBA-15, are a special class of materials that possess pores with diameters in the range of 2 to 50 nm and can adsorb large amount of water due to the amorphous surface structure and high pore volumes, exhibiting water adsorption isotherms of Type V according to the UIPAC classification [22]. These materials possess pore sizes from 2 to 15 nm, large surface areas (>500 m^2^/g), excellent thermal stability, and hexagonal pore arrangement. A large amount of water can be sorbed on them followed by capillary condensation. Basically, all the materials show high water sorption due to their mesoporosity, which is larger than that of the zeolites. The structural characteristics of ordered mesoporous matrices, such as specific surface area, the pore volume, and pore size, determine the sorption properties of the composite sorbent. The pore structure influences on the strength of the interactions between water molecules and the adsorbent sorption sites. Higher surface area means more available sorption sites in the material and indicates better diffusivity of the vapor, which is crucial for optimal mass and heat transfer. Maaz et al. [23] stated that synthesis and post-synthesis procedures could have a great influence on water sorption behavior of SBA-15, correlating mainly with the amount and type of silanol groups on the silica surface and increasing the water uptake over a broad range of relative pressure due to high micropore volume and high surface polarity. Only a few investigations have been performed on the incorporation of CaCl_2_ into ordered mesoporous silicates with mono-sized pores such as MCM-41 [24] and SBA-15 [11,25,26]. These matrices with uniform pore dimension provide an effective tool for controllable tuning of the solvation temperature of the confined salt. This temperature is higher when the salt is located in smaller pores [27]. Ponomarenko et al. prepared a composite of SBA-15 (pore diameter 7.5 nm) and 43 wt. % of CaCl_2_ by wet impregnation, showing on filling the pores with CaCl_2_ and deposition of the salt on the surface [14]. The authors showed that the Type IV nitrogen sorption isotherm of pure SBA-15 was changed to Type III, which is typical for nonporous adsorbent, indicating the collapse of the ordered matrix mesopore structure after the impregnation [28]. Ristić et al. prepared the composite combining SBA-15 matrix (average mesopore size of 10.2 nm) and 4 wt.% CaCl_2_ by wet impregnation. A low content of salt was used in order to maintain the ordered mesostructure [26]. The composite was exposed to a short-cycle hydrothermal treatment consisting of 20 cycles between temperatures of 150 °C and 40 °C at a water vapor pressure of 56 mbar, showing good initial hydrothermal stability of the composites under the operating conditions. In addition, Glaznev et al. prepared two SBA-15/CaCl_2_ composites with average mesopores: 8.5 and 11.8 nm and containing 28.2 wt.% and 29.5 wt.% of the salt, respectively. It was shown that changing the mesopore size of the matrix can influence the vapor transport [25]. Smaller salt particles in smaller mesopores sorbed water easier. The aim of both studies were to prepare the composites with the highest possible amounts of CaCl_2_ to achieve the highest energy storage capacities without detailed investigations on the structural properties and stability of these composites during water sorption, which indeed cause structural modifications.

Plugged hexagonal templated silicate (PHTS) has the same hexagonal pore arrangement as SBA-15, however, some of its cylindrical mesopores have internal porous plugs, while others are open without any major constrictions [29]. They are prepared by a modification of synthesis procedure for SBA-15, such as changing temperature and time of aging or molar ratio of reactants. The hydrothermal stability of PHTS under steaming was found to be better than that of SBA-15. In comparison to SBA-15, the micropore volume of PHTSs tend to be enhanced, hence, it can be envisioned that some of these micropores are located in the plugs. Thus, the mesoporous structure of PHTS features compartments, which appear to be accessible through the voids between constrictions or pores in the plugs [30].

Here, we present a study on the influence of water sorption on the structural properties of the composites of the plugged hexagonal templated silica (PHTS) with different contents of calcium chloride. The effect of CaCl_2_ amount on the PHTS matrix and on the water sorption capacity of composites is investigated. Water sorption properties of the composite were studied for low-temperature heat storage application, while stability of these composites was tested during 20 cycles between 40 °C and 140 °C.

## 2. Materials and Methods

### 2.1. Materials

PHTS was synthesized by modification of the SBA-15 synthesis using long-chain surfactant triblock copolymer Pluronic P123 [31] to prepare the plugged hexagonal templated silica with ordered hexagonal mesopore arrangement with average pore size lower than 6 nm. In a typical synthesis procedure, Pluronic P123 was dissolved in diluted HCl solution by stirring at room temperature until P123 was dissolved. Then tetraethylortho silicate was added, and the resulting mixture was stirred for 8 h at 65 °C, and then kept at 65 °C at ambient pressure for 16 hours without stirring. The solid product was filtered and washed repeatedly with deionized water. After drying at room temperature overnight, the product was calcined in air at 550 °C for 6 h in order to remove the surfactant. The composites were prepared by incipient wetness impregnation [32] of the PHTS matrix with concentrations: 4 wt.%, 10 wt.%, and 20 wt.% of calcium chloride. The samples were dried at room temperature overnight. The composites were denoted 4-CaCl_2_-PHTS, 10-CaCl_2_-PHTS and 20-CaCl_2_-PHTS.

### 2.2. Methods

The X-ray powder diffraction (XRPD) patterns were recorded on a PANalytical X’Pert PRO high-resolution diffractometer (Almelo, The Netherlands) with Alpha1 configuration using CuK_α_1 radiation (1.5406 Å) in the range from 0.5 to 35° 2θ with step 0.017° per 100 s using a fully opened X’Celerator detector. Morphology of the matrix and the composites was studied by scanning electron microscopy (SEM) on a Zeiss SupraTM 3VP SEM microscope (Jena, Germany). Transmission electron microscopy (TEM) micrographs were obtained on a 200-kV field-emission gun (FEG) microscope JEOL JEM 2010F (Peabody, MA, USA). Elemental analysis was performed by energy dispersiveX-ray analysis (EDAX) with an INCA Energy system attached to a Zeiss SupraTM 3VP microscope (Jena, Germany). Nitrogen physisorption measurements were performed at −196 °C on a Tristar volumetric adsorption analyzer (Micromeritics, Norcross, GA, USA). Before the adsorption analysis, the samples were outgassed under vacuum for 2 h at 200 °C in the port of the adsorption analyzer. Prior to the evaluation of textural properties of the composites the amount of the nonporous salt was taking into account which does not contribute to nitrogen adsorption to a large extent. Thus nitrogen isotherms and all specific values (surface area, pore volume) were corrected. The BET specific surface area [33], S_BET_, was calculated using the adsorption branch in the relative pressure range between 0.05 and 0.16. The total pore volume, Vt, was estimated from the amount adsorbed at a relative pressure of 0.96. The pore size distributions (PSDs) were calculated from nitrogen adsorption data using an algorithm based on ideas of Barrett, Joyner, and Halenda (BJH) [34]. The maxima on the PSD are considered as the primary mesopore diameters for given samples. Water sorption characteristics of the samples were determined by an IGA-100 gravimetric analyzer (Hiden Isochema Ltd., Warrington, UK). Water sorption isotherms were obtained at 25 and 40 °C by setting equal pressure intervals of 1.6 mbar between vacuum and 40 mbar (saturation vapor pressure of 73.8 mbar at 40 °C) with an equilibrium time of 80 s. Before adsorption measurements, the samples were outgassed to a constant weight under ultrahigh vacuum (<10^−5^ mbar) at 150 °C for 5 hours. The hydrothermal stability of the materials was evaluated with 20 cycle measurements in a helium gas flow with 75% relative humidity by varying the temperature between 40 and 140 °C at 56 mbar. The relative humidity was controlled by varying the ratio of dry and saturated helium via two mass flow controllers. The water capacity of materials was measured at the beginning and after the 20 cycles. The definition of the thermodynamic heat cycle and the calculation of the amount of heat involved are given by De Lange et al. [35]. First, the adsorption equilibrium data of each sample obtained using the IGA-100 was plotted as the so-called characteristic curve (adsorbed water uptake as a function of the adsorption potential A). The adsorption potential A is defined as: A = RTln(p_s_(T))/p (1), where R is the gas constant, T the temperature, p_s_ the saturation pressure, and p the vapor partial pressure [36]. The integral enthalpy of adsorption Q_ads_ can be calculated by the following equation: Q_ads_ = ΔH_ads_ (w_ads_ − w_des_) [kJ/kg_ads_] (2), where Q_ads_ [kJ/kg_ads_] is the enthalpy of adsorption, which can be considered as the achievable heat storage density at a material level; ΔH_ads_ [kJ/kg_water_] is the differential enthalpy of adsorption referred to the adsorbed amount of water; w_ads_ and w_des_ [kg_water_/kg_ads_] are the maximum and minimum adsorption amount of water over the adsorbent material at the given boundary conditions [6]. As the specific heat of the adsorbents is not known exactly, it was set to 1 kJ/kg K. The results were not affected by this value in a significant way [37]. The value of the differential enthalpy of adsorption was calculated through the measurement of the equilibrium adsorption curves according to the well-known Clausius-Clapeyron equation. The integral heat of adsorption was calculated for heat storage application according the literature: at a desorption temperature of 120 °C, which can be attained by solar thermal collectors. The sorption temperature was fixed to 40 °C, which is sufficient for space heating applications. The water vapor pressure during desorption and adsorption of the samples was set to 12.3 mbar (a dew point temperature of 10 °C). The difference of adsorbed water amount at 40 °C and 120 °C at 12.5 mbar is the cycle (water) loading lift of the composite.

## 3. Results and Discussion

### 3.1. Structural Properties of As-Prepared Smples

The synthesis procedure of the SBA-15 was modified in order to synthesize the PHTS matrix with average pore size of 5.7 nm with the aim to confine CaCl_2_ in the pores of this matrix. PHTS was synthesized at ambient pressure at 65 °C, while SBA-15 synthesis involved hydrothermal treatment at 100 °C [38]. It is known that aging temperature and time affects the pore size of the SBA-15, namely a higher aging temperature, increases average pore size [23]. Wetness incipient (dry) impregnation was used for the preparation of the composites.

**Figure 1:**
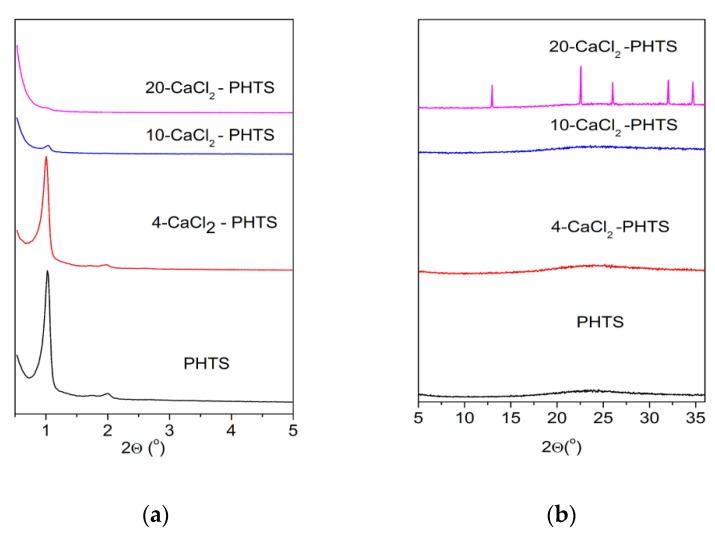
(**a**) Low-angle XRD patterns of the matrix and the composites containing different contents of CaCl_2_; (**b**) High-angle XRD patterns of the pure matrix and the composites with different amounts of CaCl_2_.

Figure 1a shows low-angle X-ray powder diffraction patterns of the PHTS and the composites containing different amounts (4, 10, and 20 wt.%) of calcium chloride. The PHTS matrix pattern illustrates three diffraction peaks corresponding to the reflections typical for two-dimensional (2D)-hexagonal pore arrangement. It can be seen that after loading of 4 wt.% of calcium chloride, three diffraction peaks were still present and their 2θ values were only slightly shifted, indicating that the channels with good order were maintained during the preparation procedure of the composite. The impregnation of larger amounts of calcium chloride of the matrix leads to the change of the diffraction patterns; only one less intensive diffraction peak was observed for the composite with 10 wt.% of the salt, while a broad diffraction peak can be seen for the composite with 20 wt.% of the salt. These results indicate the collapse of ordered arrangement of mesopores of these composites into disordered mesopore arrangement. The X-ray diffractograms recorded in the wide-angle range (5° < 2θ < 35°) are displayed in Figure 1b. XRD pattern of the composite with 4 wt.% and 10 wt.% of calcium chloride did not show any reflections of calcium chloride, which could be explained with the presence of highly dispersed calcium chloride with nanosized dimensions that are located on the surface and within the pores. Only one broad peak was observed in the range (15° < 2θ < 30°) corresponding to glass-like amorphous silicate particles. While the XRD pattern of the composite with 20 wt.% of calcium chloride showed diffraction peaks of the salt.

**Figure 2:**
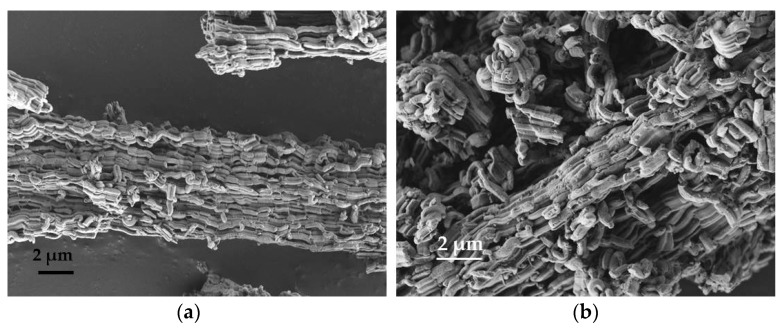

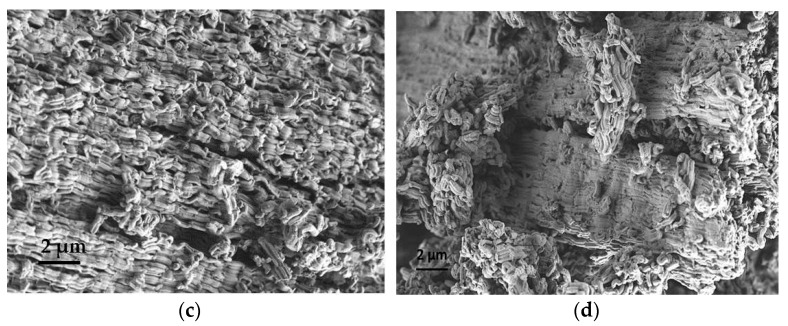
Scanning electron microscopy (SEM) images of (**a**) plugged hexagonal templated silicate (PHTS) matrix; (**b**) 4-CaCl_2_-PHTS; (**c**) 10-CaCl_2_-PHTS and (**d**) 20-CaCl_2_- PHTS.

The SEM image of the matrix presented in Figure 2a shows curved rod-like aggregates of matrix particles with a relatively smooth surface. The SEM images of the composite samples with 4 wt.% and 10 wt.% CaCl_2_ represented in Figure 2b,c, respectively, show similar morphology of the particles without any changes after the loading of calcium chloride solution. There are no observable changes of the outer surface of the composite particles. On the other hand, Figure 2d clearly shows different outer surface of the composite due to higher amount of salt (20 wt.%), which is in accordance with the high-angle XRD pattern (Figure 1b) of this sample.

**Figure 3:**
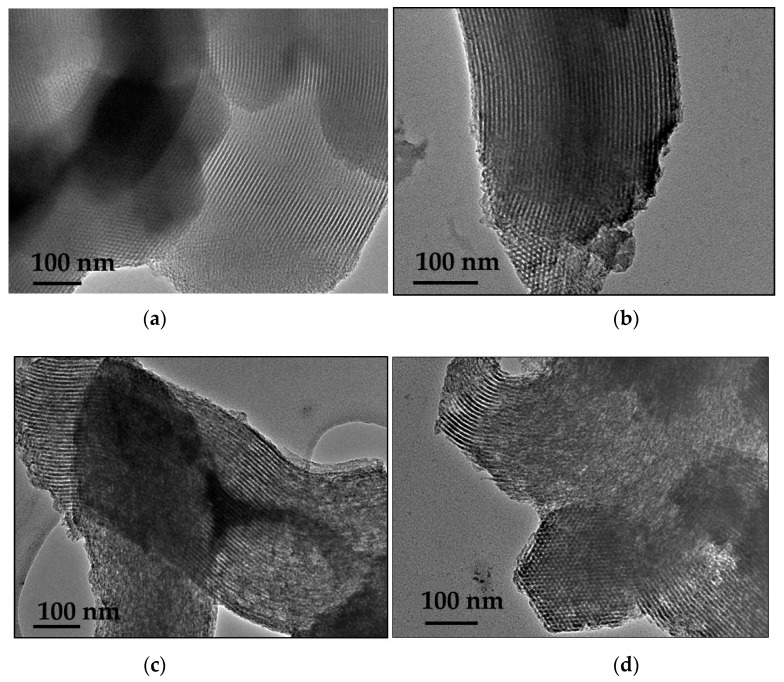
Transmission electron microscopy (TEM) images of the as-prepared (**a**) PHTS matrix, (**b**) 4-CaCl_2_-PHTS, (**c**) 10-CaCl_2_-PHTS and (**d**) 20-CaCl_2_-PHTS.

Pore arrangement of mesoporous materials in local scale was investigated by using transmission electron microscopy (TEM). TEM images (Figure 3) revealed the ordered hexagonal pore arrangement of the PHTS matrix and of the 4-CaCl_2_-PHTS, while the composites with 10 and 20 wt.% of the salt showed the presence of the disordered mesostructured. A larger amount (20 wt.%) of the salt led to the formation of disordered mesoporous structure of the composites, as can be seen in low-angle XRD pattern.

**Figure 4:**
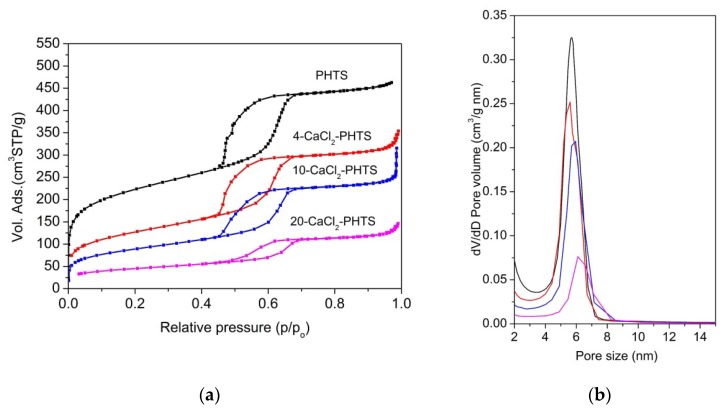
(**a**) Nitrogen sorption isotherms and (**b**) pore size distribution of PHTS and composites.

Porous structure of the PHTS matrix and all composites were examined by nitrogen sorption isotherms. Nitrogen sorption isotherms for PHTS and CaCl_2_-PHTS are shown in Figure 4a, whereas structural parameters determined on the basis of these isotherms are listed in Table 1. Due to the synthesis procedure PHTS sample exhibits sorption isotherm typical for PHTS-like material [30]. Plugged hexagonal templated silica has the same 2D hexagonal symmetry as SBA-15 with some of its cylindrical mesopores have internal plugs, while others are open as inferred from gas adsorption-desorption data. N_2_-sorption isotherms of PHTS are of type IV according to the IUPAC classification with H5 hysteresis loop [28], showing a one-step capillary condensation, two-step desorption, and an appreciable widening of hysteresis loops. The first step is similar to desorption in pure SBA-15 and is assigned to the desorption of N_2_ from the open pores; the second desorption step can be attributed to the nanoparticles (plugs) within the mesopores (the narrowed mesopores) [39]. The second step on the desorption branch indicates the existence of plugged mesopores. The presence of CaCl_2_ in PHTS leads to a marked change in the shape of the hysteresis loops, showing on partial deformation of ordered pore structure (10-CaCl_2_) into a collapse of the ordered pore arrangement (20-CaCl_2_), which finds some evidence in TEM and XRD analyses [40]. Additional hysteresis loop at relative pressure above 0.97 on sorption isotherm is present in 10-CaCl_2_ sample, evidencing the presence of an interparticle or textural porosity [41]. The increase of the amount of the impregnated salt on the matrix leads to a decreased specific surface area, total pore volume, and micropore volume. The decrease of specific surface area is related to the blockage of the smallest pores induced by CaCl_2_ impregnation. It can be concluded that CaCl_2_ nanoparticles have been dispersed inside of the micropores and mesopores of the support. Pore size distribution of CaCl_2_-PHTS materials has been determined using the BJH model, widely used for this type of samples [42]. Although this model often underestimates pore sizes [43], it is appropriate for comparative purposes. Figure 4b displays the pore size distribution determined from adsorption isotherms. As can be observed, the maximum characteristic to open mesopores of PHTS is the most intense and shows an average pore diameter of 5.7 nm.

The maximum characteristic for 4-CaCl_2_-PHTS due to CaCl_2_ nanoparticles shifted to a lower pore size value (5.6 nm), while in 10-CaCl_2_ and 20-CaCl_2_ composites they shift to 5.8 nm and 6.2 nm, respectively. The reason could be the partial destruction of mesoporous structure caused by the corrosive action of calcium chloride solution [44]. Maxima are less intensive with higher amounts of CaCl_2_.

### 3.2. Structural Properties of the Samples After Water Sorption and Cycling Test

**Figure 5:**
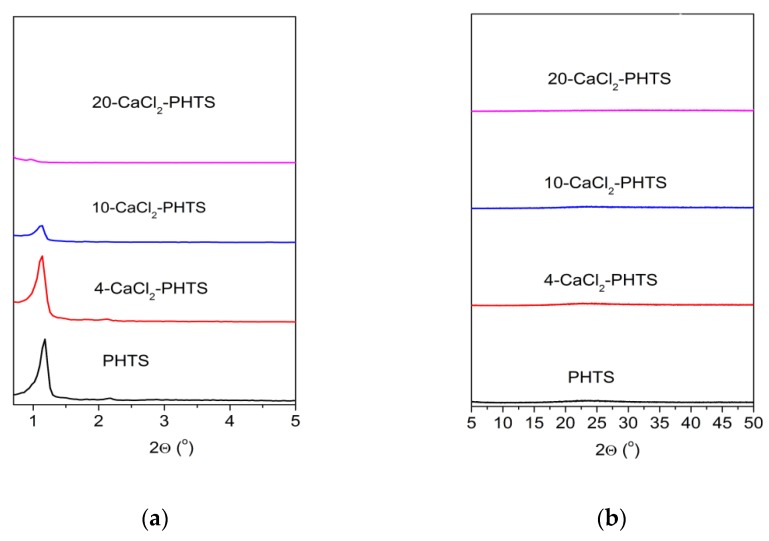
(**a**) Low-angle XRD patterns of the matrix and the composites containing different contents of CaCl_2_ after water sorption; (**b**) High-angle XRD patterns of the pure PHTS and the composites with different amounts of CaCl_2_ after water sorption.

After the measurement of water isotherms at 40 °C the low-angle XRD patterns (Figure 5) are changed, showing broader less intensive diffraction peaks. The ordered mesoporous structure of the matrix and 4-CaCl_2_ sample has been retained, as well as the disordered mesoporous structures of 10- CaCl_2_ and nonporous structure for 20-CaCl_2_ composite. High-angle XRD patterns of all samples are the same without any diffraction peaks of the salt. It seems that the salt in 20-CaCl_2_-PHTS was re-dispersed in the PHTS matrix.

**Figure 6:**
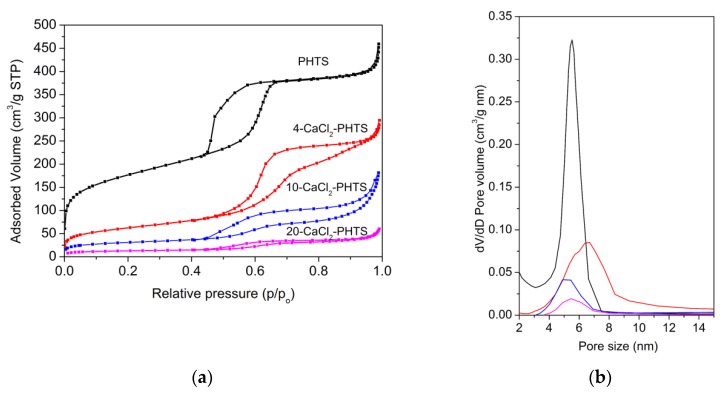
(**a**) Nitrogen sorption isotherms and (**b**) pore size distribution of PHTS and composites after water sorption.

The shape of nitrogen sorption isotherms (Figure 6) of the composites has also changed, i.e., the hysteresis loops are widening and tailing, exhibiting different types from H2 to H3 [28]. For example, the H2 hysteresis loop is related with pore blocking in silicas after hydrothermal treatment, while the H3 loop is typical of materials with slit-like pores. All isotherms show the presence of interparticle porosity. Nitrogen sorption isotherm of 20-CaCl_2_ indicates on nonporous material, which is in accordance with XRD. Structural properties are shown in Table 2. Specific surface area and total pore volume decreased after the water sorption measurement. Pore size distributions (Figure 6b) of all samples were broader. The decrease of pore sizes was observed for PHTS, 10-CaCl_2_ and 20-CaCl_2_, while significant increase in pore size was seen for 4-CaCl_2_. It may be due to the corrosion of the walls due to the salt, confined in the intra-walled pores, which interconnect the channels [45] and form larger pores. It can be concluded that the confinement of the salt was not permanent and after hydration and dehydration the salt was re-dispersed, which caused further blocking of pores (20-CaCl_2_) due to a possible agglomeration of the salt.

**Figure 7:**
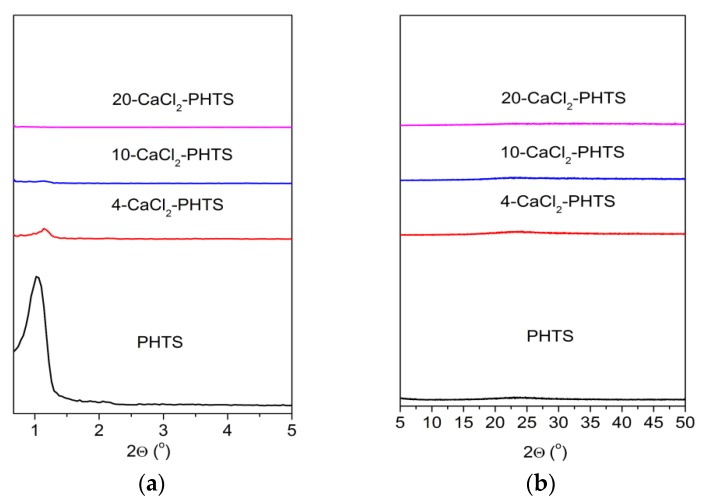
(**a**) Low-angle XRD patterns of the matrix and the composites containing different contents of CaCl_2_ after cycling test and (**b**) High-angle XRD patterns of the pure matrix and the composites with different amounts of CaCl_2_ after cycling test.

XRD patterns of the matrix and the composites after cycling test (Figure 7a) show collapse of the ordered mesostructure into disordered one for the composites containing 10 and 20 wt.% of the salt. Partial collapse is observed for the composite with 4 wt.% of the salt as well. No diffraction peaks of the salt can be observed in Figure 7b presenting high-angle XRD patterns of the composites.

**Figure 8:**
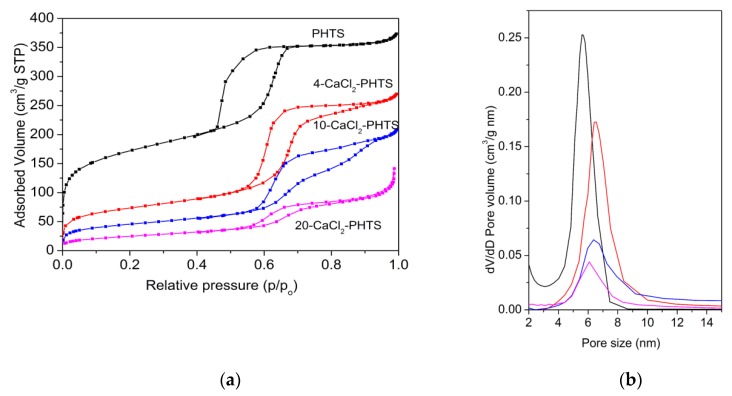
(**a**) Nitrogen sorption isotherms and (**b**) pore size distribution of PHTS and composites after 20 cycles.

It can be clearly seen that the shape of the isotherm of the matrix did not change neither after water sorption nor after 20 cycling test (Figure 8). On the other hand, the shape of the nitrogen isotherms of the composites after water sorption and cycling test is significantly different, showing strong influence of water sorption on the structure of the composite’s matrix. Porosity (Table 3) of the matrix and the composites were improved, showing the increase of specific surface area, total pore volume, and pore size of the composites. This indicates that salt was still present in the pores after the cycling test, but without salt agglomerates, thus causing further destruction of the mesopores due to corrosiveness of the salt solution and resulting in the increase of pore size of all composites. Interparticle porosity was less pronounced for the PHTS, 4-CaCl_2_, and 10-CaCl_2_ samples. Figure 8b shows broad and less intensive pore size distributions of the composites comparing to the matrix after 20 cycles.

**Figure 9:**
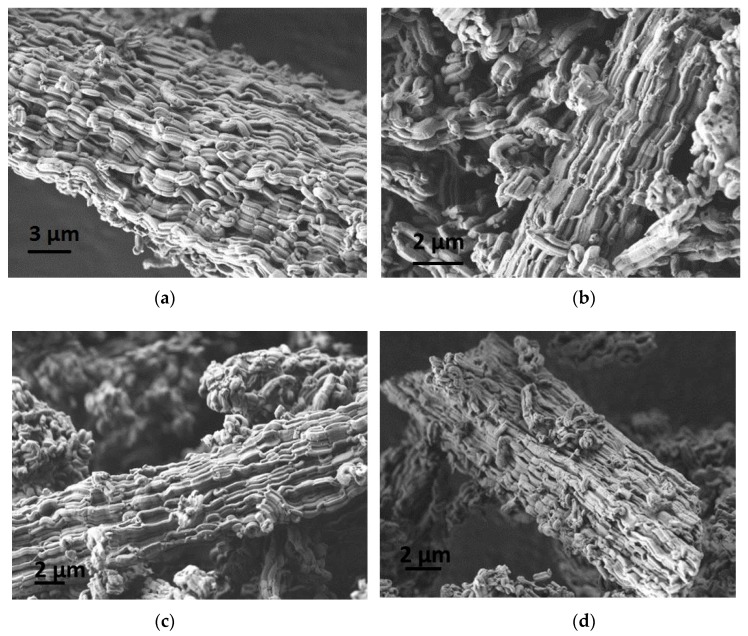
SEM images of (**a**) PHTS matrix; (**b**) 4-CaCl_2_-PHTS; (**c**) 10-CaCl_2_-PHTS and (**d**) 20-CaCl_2_-PHTS after cycling test.

SEM pictures after water sorption are similar to the pictures of the as-prepared samples. The pictures after 20 cycles are presented in Figure 9. It can be seen that morphology of PHTS and all composites did not change after the cycling test.

### 3.3. Water Sorption and Heat Storage Capacity Calculation

**Figure 10:**
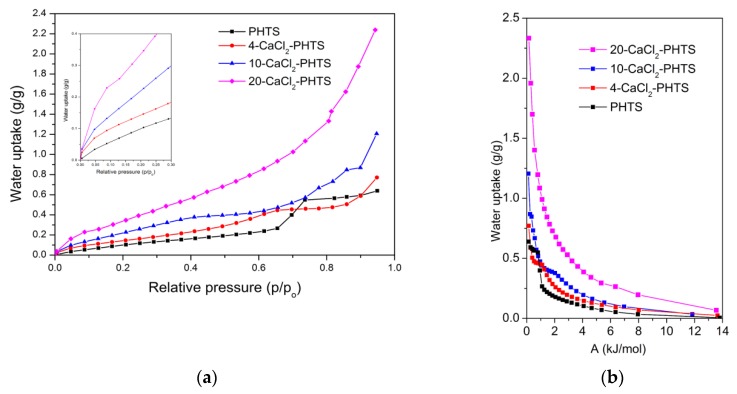
(**a**) Water uptake curves for the matrix and the composites (inset: water uptake in the range 0 < p/p_0_ < 0.30) at 40 °C; (**b**) the characteristic curves for water sorption on the PHTS and the composites.

Water sorption isotherms performed at 40 °C for the matrix and the composites are shown in Figure 10a. The ordered mesoporous matrix and the composites exhibit sorption of isotherms Type V. The water uptake curve of the matrix showed typical characteristics of weak hydrophilic or hydrophobic mesoporous materials with low sorption at low relative pressure and moderate sorption at the middle relative pressure, and sudden high water sorption at higher relative pressure [22]. The maximal water upload of the matrix was 0.65 g/g, showing the active role [14] of the matrix. A comparison of hydrophilic character of PHTS and SBA-15 from the literature [46] shows that a capillary condensation started at higher p/p_0_~0.75 for SBA-15 than for PHTS (p/p_0_~0.65), which indicates that PHTS is more hydrophilic than SBA-15. Another difference of structural property, is evident. Namely, the specific surface area, which influences hydrophilic properties and consequently Qads, of SBA-15 is 554 m^2^/g and water uptake of this material is 0.02 g_H2O_/g_sample_ in the 0 < p/p_0_ < 0.3. On the other hand the specific surface area of PHTS is much higher (810 m^2^/g) and the water uptake reaches 0.138 g_H2O_/g_sample_ in the same relative pressure range. It is well known that higher surface area means more available sorption sites in the material and indicates better diffusivity of the vapor, which is crucial for optimal mass and heat transfer. It can be concluded that lower temperature (65 °C) of aging for the PHTS preparation is beneficial for higher water uptake at low relative pressure [23]. Maximal water sorption capacities of the composites increased to 0.78 g/g (4-CaCl_2_), 1.20 g/g (10-CaCl_2_), and 2.24 g/g (20-CaCl_2_). The shape of the uptake curves was evidently changed. For a relative pressure of 0.4, the composite containing 10 wt.% CaCl_2_, showed double water sorption capacity (0.16 g/g vs. 0.38 g/g), while the composite with 20 wt.% of the salt revealed three times larger water sorption capacity than the matrix (0.16 g/g vs. 0.58 g/g). It can be concluded that the presence of calcium chloride in the matrix increased the water sorption capacity of the composites; thus, the salt content impacted the sorption performance of these composites [12]. On the other hand, the matrix of the composites with the same amount of salt had an important role as well. Namely, comparing water sorption isotherms of 4-CaCl_2_-PHTS and 4-CaCl_2_-SBA-15 [26] composites revealed differences in the range 0 < p/p_0_ < 0.4, showing higher uptake for 4-CaCl_2_-PHTS due to the preparation procedure of PHTS. Water uptake at 0.4 relative pressure of the composite with SBA-15 matrix, possessing uniform mesopores of average pore size of 10.2 nm and larger total pore volume (0.928 cm^3^/g), was lower for 0.07 g/g, while the maximum water uptake was higher for 0.10 g/g. The water uptake curve of 20-CaCl_2_-PHTS showed a plateau at 0.13 p/p_0_ due to formation of calcium chloride dihydrate [25], while for the composites with lower salt contents this plateau was not observed. The characteristic curves of the matrix and the composites, which showed the adsorbed water uptakes as a function of the adsorption potential A [47,48], are plotted in Figure 10b and are comparable with those previously reported [49].

The most relevant parameter for evaluating the effectiveness of sorbent for TES is the integral heat of adsorption. It is well known that the adsorbents with low water uptake after desorption and high water uptake after adsorption, resulting in high water loading lift to reach high storage densities, are needed for sorption heat storage. The integral heat of adsorption was calculated for the given boundary conditions for space heating [12]: adsorption temperature at 40 °C, desorption temperature at 120 °C, and a dew point temperature was set at 10 °C. The integral heat of adsorption Q_ads_ of all composites is listed in Table 4. 

The increased amount of the salt in the composites increased the calculated water loading lifts, and consequently, the energy storage capacity. A direct comparison of the energy storage capacities of these composites with other composites containing CaCl_2_ was difficult and risky, because it strongly depends on the boundary conditions. The energy storage capacity value of the CaCl_2_ (43 wt.%) encapsulated in a silica gel equaled 300 Wh/kg, considering desorption temperatures of 80 °C, adsorption temperature of 30 °C, and adsorption pressure of 12 mbar [12]. The composite of 7 wt.% CaCl_2_ confined in disordered mesoporous iron silicate matrix shows energy storage capacity of 155 Wh/kg at adsorption temperature of 25 °C, desorption temperature of 150 °C, and adsorption pressure of 12 mbar [13]. The energy storage capacity of 240 Wh/kg can be found for the aluminosilicate containing 30 wt% of the same salt at adsorption temperature of 40 °C, desorption temperature of 120 °C, and adsorption pressure of 20 mbar [15]. Higher values were achieved for MIL-100(Fe)/46 wt.% CaCl_2_ (335 Wh/kg) and MIL- 101(Cr)/62 wt% CaCl_2_ (485 Wh/kg) at desorption temperature of 80 °C, adsorption temperature of 30 °C, and adsorption pressure of 12 mbar [19].

The influence of desorption and adsorption temperature on the water loading lift was evaluated (Table 5) for the boundary conditions: desorption temperature at 120 °C, adsorption temperature at 30 °C, and due point temperature of 10 °C. It can be seen that lower adsorption temperature led to the increased water loading lifts of the composites, which corresponds to higher energy storage capacity of the composites [12], and thus promotes their applicability for low-temperature thermal energy storage.

On the other hand, a lower desorption temperature at 100 °C will decrease the performance of the composites (e.g., theirs energy storage capacities). For example, the calculated water loading lift of the 10-CaCl_2_-PHTS decreased to 0.100 kg/kg, which corresponds to the energy storage capacity of 86 Wh/kg.

Cycling stability of these composites during 20 cycles of sorption and desorption between 40 and 140 °C at 56 mbar was tested, showing a small reduction of water uptake (2–6%) after the last cycle for each composite. No leaching of the salt from the PHTS matrix was evidenced (Table 3), showing the ability of the PHTS matrix to create a stable nano-environment for confinement of calcium chloride. This confirms that these composites are promising candidates for low-temperature thermal energy storage.

## 4. Conclusions

Novel composites composed of PHTS (plugged hexagonal templated silicate) with hexagonal pore arrangement as the matrix and 4 wt.%, 10wt.%, and 20 wt.% of calcium chloride have been developed by incipient wetness impregnation. The preparation procedure of the matrix increased its hydrophilic properties, showing its active role for water sorption. Calcium chloride is shown to be located in the pores of the matrix. The presence of CaCl_2_ in PHTS leads to a partial deformation of ordered pore structure (10-CaCl_2_) or a collapse of the ordered pore arrangement (20-CaCl_2_) into the disordered mesostructure. Water sorption caused structural modifications of the composites, showing the re-dispersion and possible agglomeration of the salt in the pores, which caused some blocking of pores (lower total pore volume and specific surface areas) after hydration and dehydration at 40 °C. On the other hand, repeated sorption/desorption cycles between 40 and 140 °C at 56 mbar caused the improvement of structural properties (increase of specific surface area, total pore volume, and pore size) of the 10-CaCl_2_ and 20-CaCl_2_ composites, indicating that highly dispersed salt was still present in the pores. The increased salt content in the composites impacted the sorption performance of these composites, e.g., higher content of the salt higher energy storage capacity. An increase of desorption temperature or a decrease of the adsorption temperature increased the water loading lift and consequently the energy storage capacity, as well as the performance of the composites. The comparatively good initial stability of these composites under the operating conditions was determined without any salt leaching. These composites are promising candidates for low-temperature thermal energy storage.

## Figures and Tables

**Table 1:** Structural properties of PHTS and the prepared composites.

Sample	S_BET_ (m^2^/g)	V_tot_ (cm^3^/g)	V_mi_ (cm^3^/g)	Average Pore Size (nm)
PHTS	810	0.705	0.122	5.7
4-CaCl_2_-PHTS	461	0.492	0.037	5.6
10-CaCl_2_-PHTS	322	0.377	0.022	5.8
20-CaCl_2_-PHTS	163	0.189	0.016	6.2

Abbreviations: S_BET_, the BET surface area; V_tot_, total pore volume evaluated from adsorption isotherm at the relative pressure 0.96.

**Table 2:** Structural properties of PHTS and the composites after water sorption at 40 °C.

Sample After Water Sorption	S_BET_ (m^2^/g)	V_tot_ (cm^3^/g)	Average Pore Size (nm)
PHTS	640	0.624	5.5
4-CaCl_2_-PHTS	227	0.394	6.7
10-CaCl_2_-PHTS	133	0.195	5.2
20-CaCl_2_-PHTS	50	0.039	5.5

Abbreviations: S_BET_, the BET surface area; V_tot_, total pore volume evaluated from adsorption isotherm at the relative pressure 0.96.

**Table 3:** Elemental analysis and structural properties of PHTS and the composites after cycling tests between 40 and 140 °C at 56 mbar.

Sample After Cycling	S_BET_ (m^2^/g)	V_tot_ (cm^3^/g)	Average Pore Size (nm)	EDX Analysis (wt.%)
PHTS	620	0.560	5.7	-
4-CaCl_2_-PHTS	256	0.400	6.4	4
10-CaCl_2_-PHTS	165	0.304	6.5	10
20-CaCl_2_-PHTS	90	0.119	6.0	20

Abbreviations: S_BET_, the BET surface area; V_tot_, total pore volume evaluated from adsorption isotherm at the relative pressure 0.96.

**Table 4:** Water loading lift and the integral heat of adsorption for the composites.

Sample	∆w (kg/kg)	Q_ads_ (Wh/kg)	Q_ads_ (kJ/kg)
PHTS	0.073	71	256
4-CaCl_2_-PHTS	0.100	81	292
10-CaCl_2_-PHTS	0.142	119	428
20-CaCl_2_-PHTS	0.239	193	694

**Table 5:** Water loading lift and the integral heat of adsorption for the composites.

Sample	∆w (kg/kg)	Q_ads_ (Wh/kg)	Q_ads_ (kJ/kg)
PHTS	0.125	117	421
4-CaCl_2_-PHTS	0.150	131	472
10-CaCl_2_-PHTS	0.250	205	738
20-CaCl_2_-PHTS	0.430	333	1199
